# Successful birth after preimplantation genetic testing for rare mitochondrial DNA mutation m.10197G>A

**DOI:** 10.1038/s41439-025-00311-5

**Published:** 2025-04-01

**Authors:** Yuki Mizuguchi, Kou Sueoka, Suguru Sato, Mamoru Tanaka

**Affiliations:** 1 Department of Obstetrics and Gynecology, Keio University School of Medicine, Tokyo, Japan; 2 Graduate School of Public Health, Shizuoka Graduate University of Public Health, Shizuoka, Japan; 3 Center for Medical Genetics, Keio University School of Medicine, Tokyo, Japan

**Keywords:** Genetic testing, Infertility

## Abstract

Here we report the first successful birth after preimplantation genetic testing for m.10197G>A mutation, a rare variant responsible for Leigh encephalopathy. Preimplantation genetic testing diagnosed the embryo with a mutant load of <5%, and transfer resulted in a live birth. The mutant load of embryos diagnosed in this case was skewed to the extremes. Skewed segregation patterns have been observed in common mutations, but this case suggests that the same phenomenon may be seen in this rare mutation.

## Data report

Mitochondrial DNA (mtDNA) disorders are usually heteroplasmic, with cells harboring both wild-type and mutated mtDNA. They may become apparent once the number of affected mitochondria reaches a certain level. The threshold varies from tissue to tissue, and the percentage level of mutant mtDNA (mutant load, ML) also varies between and within individuals, making it difficult to establish a diagnosis. This is why there is no curative treatment currently available. A great deal of attention has been focused on the field of assisted reproductive technology, and preimplantation genetic testing (PGT) has emerged as an effective preventive option to identify embryos with MLs below disease-causing thresholds.

Not all mtDNA disorders are suitable for PGT, but studies on some of the common mtDNA mutations have shown a correlation between mtDNA heteroplasmy levels in the blastomeres and those in tissues throughout embryo–fetal development^1–3^. Successful cases of PGT for these mutations, especially m.8993T>G mutation, have been reported to lead to the birth of a healthy baby^1–4^.

The m.10197G>A mutation (NC_012920.1:m.10197G>A) is a rare pathogenic mutation of Leigh encephalopathy (LE). In a report of three different families with m.10197G>A, all affected children had severe LE with onset in infancy, with most dying within the first 1–2 years of life^5^. Studies on Japanese patients with LE have reported the rarity of this variant in Japan and that LE in *MT-ND3* gene is often severe^6,7^. Here, we report the first successful PGT performed for the m.10197G>A mutation.

**Fig. 1:**
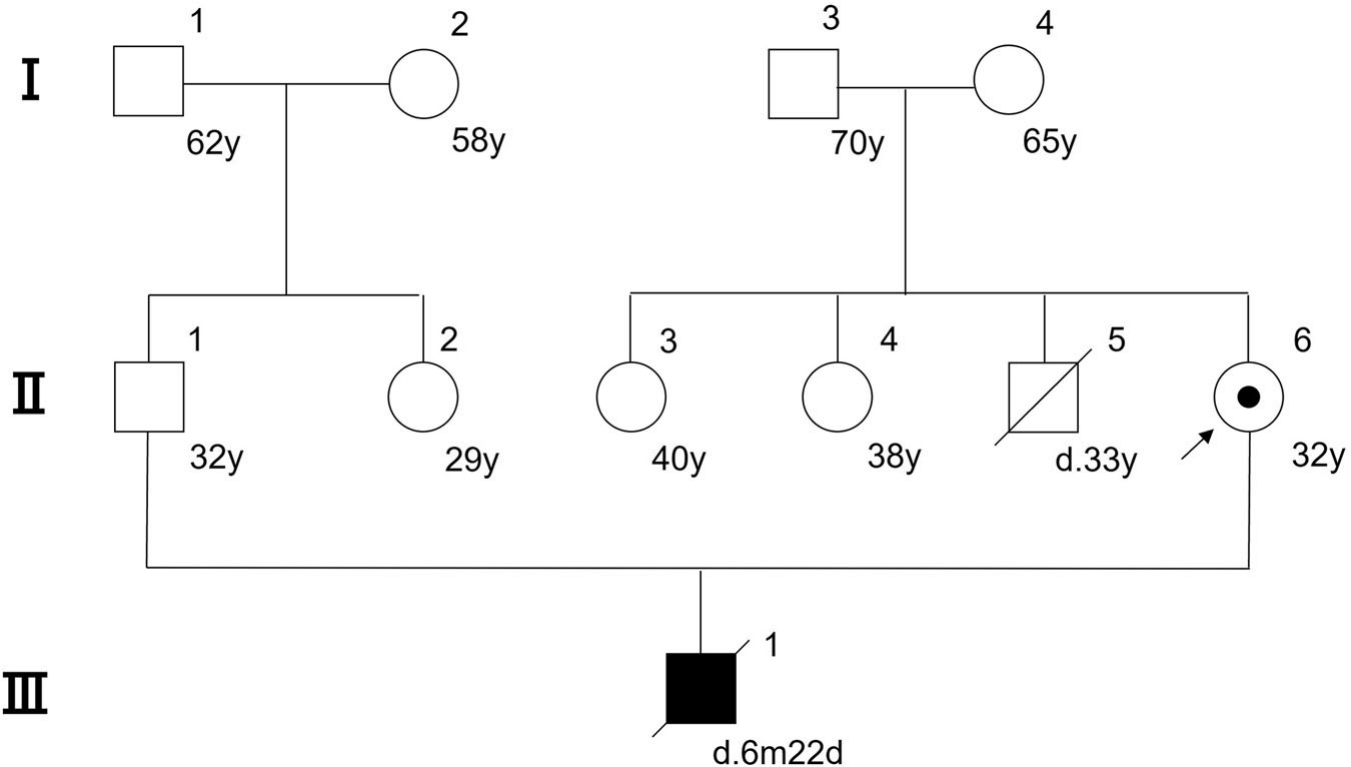
Pedigree of this study. The proband is individual III.1. Individual II.6 is the carrier and the client of this study.

A 32-year-old female carrier of the m.10197G>A mutation in the MT-ND3 and her husband requested PGT to fulfill their desire for a healthy child. Their son (proband) was born at term following an uneventful pregnancy of healthy unrelated parents. From soon after birth, he had feeding difficulties and weight gain was poor. Psychomotor retardation became apparent by 4 months, and at 6 months of age, he was hospitalized for respiratory insufficiency. LE was suspected from these symptoms, and analysis of mtDNA mutation was carried out using peripheral blood. The results confirmed homoplasmy of a point mutation in the ND3 region, and a genetic diagnosis of LE was made. He died 7 months after birth owing to LE. The ML of the son and the mother was >99% and 11%, respectively. The pedigree is shown in Fig. 1. After thorough genetic counseling for alternative methods of reproduction including PGT, informed consent was obtained. The study was approved by both ethical committees of the institute and Japan Society for Obstetrics and Gynecology.

**Fig. 2:**
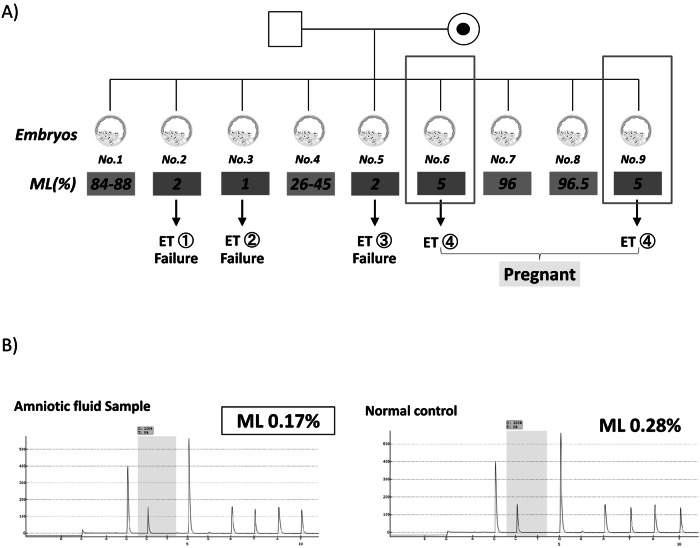
Clinical course. **A** Nine embryos were biopsied. Apart from one embryo that showed an intermediate ML, a highly skewed distribution of embryos harboring either homoplasmic mtDNA mutation or normal mtDNA is seen. ET, embryo transfer. **B**, Pyrosequence analysis of amniotic fluid sample. ML analyzed from the amniotic fluid sample was 0.17%, which was lower than the normal control (0.28%).

Controlled ovarian stimulation was performed using the GnRH agonist flare protocol, and mature oocytes were retrieved and fertilized via intracytoplasmic sperm injection. The embryos were cultured to day 5 blastocyst stage, and five to six trophectoderm cells were biopsied from each embryo for analysis. DNA was extracted and mtDNA mutation analysis was done by pyrosequencing assay. In brief, extracted DNA as a template (10–20 ng) was amplified using a PCR kit (PyroPCR; Qiagen) and thermal cycler (GeneAmp PCR System 9700; Applied Biosystems). The amplified mtDNA fragments were sequenced using a pyrosequencing instrument (PyroMark Q24 Advanced; Qiagen) with a PyroMark Q24 Advanced Reagents kit (Qiagen) according to the manufacturer’s instructions. The obtained data were analyzed using PyroMark Q24 Advanced Software (ver. 3.0.0; Qiagen). As there was no previous report concerning PGT for this mutation, we referred to a systematic review^8^ and decided that embryo with ML <18% would be feasible for embryo transfer. Approval from the ethical committee was obtained with <18%, but the couple chose a stricter threshold of <11%, based on the mother’s asymptomatic status, to ensure a higher likelihood of a healthy outcome. Apart from the ML, embryo selection was based on embryo score criteria^9^. From three PGT cycles, 15 oocytes were retrieved and 9 embryos were biopsied. Two out of the nine had >95% ML and were predicted to be affected. Five embryos with an ML of <5% were determined to be unaffected and eligible for transfer (Fig. 2A). Viable pregnancy was achieved on the fourth attempt with a double-embryo transfer. Amniocentesis was performed at 15 weeks of gestation. The ML analyzed from the amniotic fluid sample was 0% and reconfirmed the PGT diagnosis (Fig. 2B). She delivered a healthy girl weighing 2,854 g after a spontaneous vaginal delivery at 39 weeks of gestation. The child is now 3 years of age with normal growth and has not shown any symptoms related to LE.

This case demonstrates both the potential and the challenges of PGT for mtDNA disorders. One major difficulty lies in the inherent variability of mtDNA inheritance and heteroplasmy, which complicates predictions of phenotype from ML. For the m.10197G>A mutation, no prior studies had established a reliable ML threshold for disease manifestation, although a previous report suggested symptomatic cases with MLs as low as 67% (ref.^5^). The couple’s decision to set the threshold below the mother’s ML provided a conservative safeguard, maximizing the chances of obtaining an unaffected child.

A noteworthy finding in this case was the discrepancy between the ML in the transferred embryos (~5%) and almost 0% analyzed by amniocentesis. This divergence raises questions about whether it reflects biological changes in mtDNA heteroplasmy during development or the limitations of pyrosequencing analysis, particularly at very low ML levels. Trophectoderm biopsy assumes an even distribution of ML across the embryo, but evidence of mosaicism in other contexts, such as chromosomal aneuploidy, suggests that this assumption may not always be valid. Further research is needed to explore the dynamics of mtDNA distribution during development and its impact on phenotype prediction.

Another interesting point in our case was that the MLs of the embryos that were biopsied were skewed to the extremes, where the offspring will be either affected or unaffected. There are no previous studies concerning PGT for this mutation, but the distribution of ML seen in the embryos in this case is suggestive of a skewed pattern in oocytes and embryos. A similar phenomenon has been reported for m.8993T>G mutation^4,10,11^. Whether this phenomenon is something that is unique to the m.10197G>A mutation as well requires the accumulation of more cases.

This is the first successful PGT for the m.10197G>A mutation and the only report suggesting the possibility of a skewed segregation pattern in the inheritance of this rare variant. We conclude that PGT can be a useful reproductive option for carriers of rare mtDNA mutations.

## HGV datbase

The relevant data from this Data Report are available via the Human Genome Variation Database at 10.6084/m9.figshare.hgv.3491.
